# Infantile Scurvy Involving the Hip Joint1Read at the forty-second annual meeting of the Medical Association of Central New York, held at Auburn, October 19, 1909.

**Published:** 1910-02

**Authors:** Nathan Jacobson

**Affiliations:** Syracuse, N. Y.; Professor of Surgery, College of Medicine, Syracuse University; Surgeon to St. Joseph’s Hospital


### Infantile Scurvy Involving the Hip Joint[1]

By NATHAN JACOBSON, M. D., Syracuse, N. Y.

Professor of Surgery, College of Medicine, Syracuse University; Surgeon to St. Joseph's Hospital.

I DESIRE to call the attention of the association to a condition occurring in infancy which I believe is more frequently overlooked than is any other pathologic change occurring in early childhood. I refer to infantile scurvy. Without doubt, it is of more frequent occurrence since the introduction of patent foods as the main article of the infant's dietary.

The frequency with which it is unrecognised is referred to by Morse in a paper published two years ago. In thirty cases seen by him in consultation the condition has been recognised but five times. In the others it had been mistaken for rheumatism, difficult dentition, Pott's disease, hip joint disease, periostitis, gout, nervousness, infantile paralysis, syphilis of the cord, strain or injury, tuberculous gumma of the eye, acute nephritis, tumor of the bladder, uric acid and arsenical poisoning.

Von Stark in his able article on this subject in the very elaborate treatise on the diseases of children by Pfaundler and Schlossman, refers to the condition as having been mistaken for periostitis, osteomyelitis and osteosarcoma, and because of the mistake in diagnosis operations had been undertaken for the removal of these serious conditions which were presumed to but did not exist.

1. Read at the forty-second annual meeting of the Medical Association of Central New York, held at Auburn, October 19, 1909.

It is surprising that this condition has only recently received proper consideration. In an article by Still, which appeared in the *British Medical Journal* of July, 1906, he quotes from the English translation of Glisson's work on rickets and infantile scurvy, published in 1651, the original work having appeared in Latin one year earlier. Not only was this the first published work upon rickets, but in it the occasional association of these two conditions is clearly set forth and the description of infantile scurvy so well portrayed, that one can but marvel at the accuracy of the observations made by the author over two hundred fifty years ago. For more than two centuries this work was entirely lost to sight, nor was there anything written upon the subject again until Moeller published an article upon "acute rickets" in 1859. This did not attract much attention, neither did a minute description in the *London Lancet* in 1878 by Cheadle. The epoch making paper however appeared in 1883, written by Barlow and was entitled "On cases described as acute rickets which are probably a combination of scurvy and rickets, scurvy being the essential and rickets a variable element." This paper was such a very exhaustive clinical and pathological study of the disease, that since that date infantile scurvy has been designated "Barlow's disease." The first reference made to it in any textbook in the English language was in that of Osler, published in 1892. That this condition was so generally overlooked for two centuries, and since its recent resuscitation in medical literature has been so frequently mistaken for other conditions, is my excuse for presenting the subject for your consideration.

As a text for what I have to say I beg to refer to two cases seen in January of the present year. The first was a child a little less than a year old, whom I saw in consultation with Dr. Coe. Two years before, I had operated upon the sister of this child for the removal of tubercular glands of the neck; otherwise the family history was negative. The little fellow had always been weakly and not until he was seven months old was he able to sit up alone. Prior to that time his back had been too weak to support him, although he was fat and when lying down gave one the impression of being well. On Thanksgiving Day, 1908, he fell from a chair striking upon his left hip. From this date he suffered from severe pain in the left hip and thigh, although there had been no evident bruise. The extremity could not be moved nor even touched without causing great pain. I learned in the course of my study of the case, that for several months prior to the receipt of this injury the child's gums had repeatedly bled. He had also had frequent discharges of blood with the movements of his bowels.

Upon examination I found the child apparently well developed but pale and fretful. He would begin to cry as soon as he was lifted out of bed. Upon comparing the two extremities there was no apparent wasting nor deformity. The left leg and thigh were held in a flexed position. The right leg could be freely moved without causing any pain and no other part of the body was evidently affected. However, the slightest manipulation of the left lower extremity caused him to cry out. There were no changes in the gluteal cheek nor in the creases between the nates or below the buttock. There was, however, marked fixation of the hip associated with exquisite tenderness. The gums were found spongy and bled easily. The child had been brought up on a patent food known as "Lane's food."

Recognising the condition to be one of scurvy, the dietary was changed along the lines to be suggested when we consider the subject of treatment. Because of the very acute and evidently marked disturbance in and about the joint, it was deemed wise to secure absolute rest for it and therefore weight extension was applied for a time. Immediaitely upon correcting the diet and giving rest to the joint, improvement followed and within a short time the child was free from pain, slept naturally, and began to improve in color and vigor. Considerable care had to be exercised in the regulation of his diet as his digestive apparatus was very easily disarranged.

I looked up the patient about two weeks ago and found that he had become quite a sturdy little fellow. He had cut all but his last molar teeth. The gums were in excellent condition; his complexion was ruddy. Three months before he had commenced to walk and now was running about. In every way he presented the appearance of a vigorous child.

Ten days after my consultation in the case of this child, I was asked to see another by Dr. Curtin. The first evidence in this case of disturbance had been observed during the early days of November, 1908. In attempting to put on the child's shoes he would cry and from this time on there was evidently marked pain in his lower extremities. This was more pronounced in the right than the left. While affecting both hip joints there was also some disturbance in each knee. It was impossible to raise the child out of bed because with each effort to lift him he cried pitifully. He had grown very pale and the parents had become alarmed because a cousin of the child was afflicted with hip joint disease. Going into the history more fully, I learned that the child had had nose bleed frequently for at least six months, and that for the two months prior to my visit to him there had been daily hemorrhages from the gums. Examination did not disclose any swelling or marked change about the knee joints except that they were very sensitive. Each hip joint, and particularly the right one, was quite rigid and tender. There was no wasting of the thighs or buttocks and no indication of muscular atrophy so characteristic of hip joint disease. He cried most lustily when an effort was made to move his thighs. The gums were swollen and spongy, especially in the neighborhood of the upper central incisors. This child was being fed upon peptogenic milk powder. After the change in his dietary and without any medication or attention to the local condition the little patient improved at once, and within a week was entirely free from pain and discomfort.

Our first case presents some interesting features. While the child gave evidence of faulty nutrition during the first seven months of his life, the acute disturbance was precipitated by a fall which he sustained when he was about eleven months old. The resulting manifestations were so marked that they could readily have been mistaken for a pure traumatism. However, this is in keeping with the history of infantile scurvy, inasmuch as one of its characteristics is that slight injuries are apt to provoke serious disturbance. The history of tubercular glandular disease in a sister, made one apprehensive that his hip trouble might be of this type. The limitation of the disturbance to a single hip is exceedingly rare in scurvy, and in this respect strikingly simulated true hip joint disease. Had the latter condition existed, however, we should have had apparent lengthening of the extremity with abduction of limb and eversion of the foot, instead of mere flexion. We should have found too, marked wasting of the muscles of the thigh and those of the gluteal region. The gluteal fold should have been effaced and the internatal crease displaced. While fixation of the joint would have been present, the pain and tenderness would not have been by any means as great. Moreover, in hip disease the pain is not constant and is not evinced on slight motion, but begins to be pronounced when on flexing the thigh the limit of mobility is reached.

It is certainly unusual for infantile scurvy to select a single joint as its storm center, and still more so to have this the hip joint. Ordinarily the affected site is the lower end of the femur and not the upper. However, there are cases on record where hemorrhage into the hip joint has occurred. Still, in the article previously referred to, makes the following statement: "In a drawing preserved at the Children's Hospital, Great Ormond street, a hemorrhage is shown under the synovial membrane of the acetabulum in a case of infantile scurvy." Ordinarily, the hemorrhagic disturbance is outside of the hip joint, and when affecting the upper enc of the femur extends down the shaft for a distance from the uppe: epiphysis. Frequently as a result of traumatism in infants affected with scurvy, fractures or separation of an epiphysis may result. Had such been the case with this patient this would have been apparent in the position of the extremity as well as in the character of its mobility. It had been suggested in this case that the child might be suffering from a rheumatic condition. It is generally conceded, however, that articular rheumatism does not occur in children of this age.

Our second case presents a greater number of the ear marks of infantile scurvy as it is usually encountered. The involvement of the extremities was more widespread and although the right hip was particularly involved the left was also to some degree, as was each femur at its lower end.

In considering this subject the features most to be emphasised are those which concern the diagnosis, for when the condition is correctly interpreted the treatment is exceedingly simple, and the cure usually prompt and satisfactory.

The epiphyseal areas in children are particularly vulnerable. Following slight traumatisms serious infections are apt to occur, such as acute osteomyelitis or epiphysitis. A study of the clinical history of infantile scurvy teaches that it does not occur suddenly nor that its course is rapid. Even when in scurvy a traumatism may have provoked a marked aggravation of the manifestations or awakened disturbance at a fixed point, it will be found that the child has for a long period shown evidence of faulty nutrition; that hemorrhage in some form has occurred either from ths gums or bowels; or still more frequently has hematuria been evident. In some cases this latter form of bleeding persists for a long time before the charactertistic signs of scurvy appear in the extremities. Moreover, with infantile scurvy the symptoms of an infectious condition are absent. The temperature is normal or slightly elevated; when swelling appears it is of limited extent and never so acute or widespread, nor does the clinical history cover but a few days, as is the case in an infectious process involving a bone or an epiphysis. The changes in the blood are slight in infantile scurvy. There is usually a reduction in the percentage of hemoglobin, and when leukocytosis is present there is an increase in the mononuclear cells. In an infectious disturbance the leukocytosis is pronounced, and instead of an increase in the mononuclear cells there is marked increase in the percentage of the polymorphonuclear ones.

There hardly seems to be any excuse for mistaking infantile scurvy for sarcoma, and yet the mistake has been made and children have been subjected to operation for the removal of a supposed tumor, when the condition has been simply that of scurvy. It should not be necessary to dwell upon the differential diagnosis between a tumor of this type and the condition under consideration. In the case of sarcoma a tumor appears in the shaft or extremity of the bone which is of comparatively slow growth and is of definite form. The surface veins are prominent and the swelling is elastic; or it may present crackling walls. It is not tender nor is there a marked degree of pain on motion; but it has periods of pain which come on during the night or at least are quite independent of any mobility of the part.

It is to be remembered that in protracted cases of infantile scurvy marked disorganisation of an epiphysis may occur, and that fracture or separation at this point may result from violent manipulation or undue force.

Infantile scurvy is rarely encountered before the sixth month of life and occurs most frequently from this to the twelfth month; three-fourths of the cases occur during this period. If there be any doubt as to the nature of the trouble the patient can be subjected to a dietetic test. Still makes the statement that he would lay it down as a rule that if the antiscorbutic diet has produced no definite improvement within four days, the diagnosis of scurvy should be questioned.

A few moments need only be given to the all-important subject of dietetic treatment. In these days when the artificial feeding of infants is so general, it is perhaps surprising that we do not more frequently encounter this condition as a result of faulty nutrition. That there seems to be in some children a predisposition to scurvy is evident, inasmuch as it has occurred in the case of twins fed alike, that one has developed scurvy while the other has not.

The disturbance is not only attributed to the use of patent foods, but investigation has shown that heat may so alter the chemical constituents of milk, as to favor the production of scurvy. Practically, no cases are on record of scurvy occurring in breast fed children. Moreover, it seems that but few cases have followed the use of good cow's milk when used unboiled or but slightly warmed. Long continued boiling or the sterilisation of milk seem to be contributing factors in the production of infantile scurvy. The addition of patent foods to milk which has been overheated, or to so-called condensed milk seems to particularly favor its development. On the other hand, it is well established that if to the child's diet of milk there be added fruit juices, fresh meat juice and potato, that scurvy can be avoided and if present will promptly disappear. Orange and grape juices are particularly helpful. The former can be administered three to four times a day in teaspoonful quantities an hour before the ordinary feeding of the child, or a smaller quantity of the fresh juice of grapes will accomplish the same purpose. Beef juice pressed out of the fresh beef, and not taken from meat which has been roasted, is beneficial but not quite as efficient. Our English confreres are very fond of preparing a cream, by adding the outer mealy portion of a boiled or steamed potato to milk, being careful to make a perfectly smooth mixture.

It seems to me that this subject should particularly interest this association composed as it is so largely of general practitioners. At the same time it should prove attractive, alike, to the internist and the surgeon, not only from a diagnostic standpoint but because it yields so readily to proper treatment, while the failure to recognise it may lead to very serious consequences.

430 S. Salina Street.

